# Supplementary material for: Investigating the basis of lineage decisions and developmental trajectories in the dorsal spinal cord through pseudotime analyses
Source: Development. 2024 May 30;151(10):dev202209. doi: 10.1242/dev.202209 (PMC11166460; doi:10.1242/dev.202209)
Supplement: Supplementary file 1 [file develop-151-202209_review_history.pdf]

Investigating the basis of lineage decisions and developmental trajectories in the dorsal spinal cord through pseudotime analyses

Sandeep Gupta, Eric Heinrichs, Bennett G. Novitch and Samantha J. Butler
DOI: 10.1242/dev.202209

Editor: François Guillemot

Review timeline

Original submission:	24 July 2023
Editorial decision:	30 August 2023
First revision received:	19 March 2024
Accepted:	18 April 2024

Original submission

First decision letter

MS ID#: DEVELOP/2023/202209

MS TITLE: Investigating the basis of lineage decisions and developmental trajectories in the dorsal spinal cord through pseudotime analyses

AUTHORS: Sandeep Gupta, Eric Heinrichs, Bennett G Novitch, and Samantha J Butler

I have now received the reports of three referees on your manuscript and I have reached a decision. The referees' comments are appended below, or you can access them online: please go to BenchPress and click on the 'Manuscripts with Decisions' queue in the Author Area.

As you will see, all the referees express great interest in your work, but they also have significant criticisms and recommend a substantial revision of your manuscript before we can consider publication. In particular, they would value adding a comparison of the pseudotime analysis of the in vitro differentiated dorsal interneurons with a similar analysis performed on in vivo embryonic spinal cord scRNA-Seq data. If you are able to revise the manuscript along the lines suggested, which may involve further experiments, I will be happy to receive a revised version of the manuscript. Your revised paper will be re-reviewed by one or more of the original referees, and its acceptance will depend on your addressing satisfactorily the reviewers' major concerns. Please also note that Development will normally permit only one round of major revision.

If it would be helpful, you are welcome to contact us to discuss your revision in greater detail. Please send us a point-by-point response indicating your plans for addressing the referees' comments, and we will look over this and provide further guidance.

Please attend to all of the reviewers' comments and ensure that you clearly highlight all changes made in the revised manuscript. Please avoid using 'Tracked changes' in Word files as these are lost in PDF conversion. I should be grateful if you would also provide a point-by-point response detailing how you have dealt with the points raised by the reviewers in the 'Response to Reviewers' box. If you do not agree with any of their criticisms or suggestions please explain clearly why this is so.

Reviewer 1

Advance summary and potential significance to field

© 2024. Published by The Company of Biologists under the terms of the Creative Commons Attribution License (https://creativecommons.org/licenses/by/4.0/).

This study by Gupta et al builds on this group's scRNA-seq data published in Cell Reports 2022 that was based on in vitro induction of mESCs to spinal sensory neurons generated in the dorsal neural tube. The previous study reported the optimized protocols for generating dorsal interneuron subtypes dI1-dI6 from mESCs, and scRNA-seq to conclude how they are indistinguishable from in vivo derived dI neurons. This current study uses these data for pseudotime analysis to infer the developmental trajectories from progenitor dP states to the dorsal interneurons dI. They combine the scRNA-seq data from day9 from 2 protocols distinguished by +/- BMP4 to preferentially give dI1-3 and dI4-6, respectively. The developmental trajectories describe branch points reflecting dI lineage decisions in this in vitro system. Known lineage marker genes are used to guide the analysis and then the co-expressed genes in the clusters identify genes not previously used to define the lineages or branch points. Expression of nine of these are shown by in situ hybridization in embryonic neural tubes to validate expression is in regions consistent with the interpretation of the pseudotime analysis. A potentially new subset or endpoint of the dI5 lineage is suggested. This pseudotime analysis and interpretation provides a valuable resource and characterization of an in vitro differentiation paradigm for generating mouse spinal sensory neurons from mESCs. A concern is that conclusions here are used to infer the same thing is happening in vivo, while their strength is defining what is happening in the in vitro system. Similar to previous work from this group, it would be valuable to perform the same pseudotime analysis on mouse embryonic neural tube scRNA-seq data to distinguish and/or confirm their conclusions on developmental branch points and lineage relations from the in vitro system. Without this comparison, it should be made clear (even in the title) that the analysis here is on the in vitro differentiation system.

Comments for the author

Specific comments.
1) As above, are the genes in the branch points and the branch points themselves, the relationship of dI2/3/4, and the new endpoint for dI5 also detected in vivo? A thorough handling of the in vitro scRNA-seq data is included and there are interesting points being made about transitions during dI development. It is easy to forget that these are made on the in vitro system so some rules may not hold in vivo. It should be clear what does hold in vivo and what happens in the cultures.

2) Fig 2E is a little misleading (not purposely I'm sure, but from the point of view of a naïve reader) in that it suggests the genes are specific to the different populations but a closer look at Fig S2 suggests that is not that simple (example: Sox4, highlighted in dI5 but is in all the lineages). It is recommended that this point be more explicit when discussing Fig. 2E in describing how these genes are identified and what they mean. Overall however, the way the data are presented for the different dI lineages and the heatmaps provides excellent visuals for what is going on within a given lineage.

3) In the section 'Validation of putative transition state markers in vivo', it would be helpful to put the transitional cluster #s at the beginning sentence where the nine genes came from that were chosen for validation. Along these lines, a supplementary table should be provided showing the top genes (50?, 100?) in each cluster as defined in Fig. 1E since these clusters are used throughout the study. The shinyApp is a useful tool to look through the data but a file with the genes defining the clusters is also needed.

4) Not all known lineage markers in vivo appear in the in vitro data. One that is discussed a little is Phoxa2. It is suggested the expression is too transient to show up in the data. Another factor notably absent is Ptf1a that is required for the dI4 lineage in vivo. Are Phoxa2 and Ptf1a not in the data because they are too transient? Technical artifacts of scRNAseq? Or is this a difference between the in vitro neural induction versus in vivo development? Clarification through analysis of scRNA-seq from in vivo neural tubes would potentially resolve this.

Additional Minor comments:
5) There is no real discussion of what is going on in the progenitor clusters as visualized in Fig. 2C. Are there any insights to come out of this cloud of cells that say anything about progenitor diversity?

© 2024. Published by The Company of Biologists under the terms of the Creative Commons Attribution License (https://creativecommons.org/licenses/by/4.0/).

6) Data presentation in figure 3 would be enhanced with co-expression/adjacent known markers for the different populations. Currently, the interpretation of the in situ data must remain imprecise and should be reflected in the way it is described.

7) Suggestion: Figure 5 shows nice co-staining of Sncg with Lmx1b and Pax2. The high mag images shown for the triple are clear. If possible, it is suggested to do the same for Sncg/Lmx1b and Sncg/Pax2 rather than the lower mag images.

8) On pg 16 in the discussion, it is not clear how the few sentences on Tfap2b and Ptf1a fits with the point of the paragraph. Maybe integrate or delete?

Reviewer 2

Advance summary and potential significance to field

This study is an extension of reported studies from these investigators, regarding dorsal interneuron development gained from an in vitro differentiation scheme. By compiling peudotemporal trajectories, they were able to gain new insight into the lineage relationship and developmental control of the dorsal horn interneurons and their progenitors. Some of the observations from in in vitro dataset were confirmed via in vivo expression analyses. However, a few issues need to be addressed.

1) Is this surprising for inhibitory DI4 neurons and excitatory dI2 and dI3 to share the same lineage relationship during early development?

2) Related to the above points, the authors try to claim that at later stages dI2-6 neurons start to share similar transcriptomes again. This again appears not to make much sense since you are talking about excitatory (DI2, DI3, dI5) versus inhibitory neurons (dI4, dI6). It is possible that this in vitro differentiation protocol may not capture the later terminal differentiation seen in vivo. Can the authors comment on the available transcriptomes of adult dorsal horn interneurons that correspond to these embryonic dorsal horn interneurons? The authors need to have some serious discussions on these points.

Comments for the author

see above

Reviewer 3

Advance summary and potential significance to field

This study adds little to what is already known about neuronal specification in the dorsal spinal cord, and it has multiple weaknesses that need addressing. In particular, the proposed trajectories require further analysis and validation. A major rewrite will also be required before it is suitable for publication.

Comments for the author

The authors have used an in vitro ES cells differentiation protocol to analyze the developmental trajectories of dorsal spinal cord progenitors . They draw a number of conclusion from their bioinformatic analysis, namely that the dI5 interneuron progenitor population, followed by dI1 progenitors are the first to branch off a common dorsal progenitor lineage, with dI2/3/4 progenitors forming a common late lineage that then separates into three lineages. The authors identify a number of genes that mark this transition, although the rationale for choosing these genes and the function of these genes is in many cases is not adequately discussed. There is some concern with the the proposed trajectories for dI1-dI6 neurons, and how these trajectories comport with previously published in vivo analyses at these early embryonic stages. Further in vivo validation of these trajectories is needed, potentially by comparing their in vitro analysis with a

© 2024. Published by The Company of Biologists under the terms of the Creative Commons Attribution License (https://creativecommons.org/licenses/by/4.0/).

similarly performed dorsal spinal cord single cell RNASeq analysis at times spanning the early differentiation of dorsal spinal cord cell types, i.e. E9-E11.

I would also argue for a far more scholarly reading and citing of the literature as many critical studies were not cited. Moreover, in some instances these studies suggest alternate trajectories and developmental decision points. For example, the functional analysis of Ptf1a (Glasgow et al., 2005) argues for a common progenitor for dI4 and dI5 neurons, and there is evidence that at E10.5 Lmx1b dI5 neurons are born after Pax2+ dI5 neurons have already been generated. There is evidence for Notch signaling controlling the fate of dI4 versus dI5 neurons. While the findings in this study may be correct, this needs to validated and then discussed in the context of the published literature. For example, there needs to be a fuller discussion of genes such as Atoh1, Ngn1/2, Olig3, Ascl1, Lbx1, Ptf1, Notch and Gbx1/2 that have demonstrated roles in dorsal cell specification

I also have some concerns with the analysis of Sncg. Ther authors show expression at E11.5, which is quite late, as dI5 neurons are first generated at E10-E10.5. Better panels showing co-expression with Lmx1b is needed (Figure 5) as it is hard to ascertain whether these two genes are co-expressed in dI5 cells. The expression of Scng from the Allen sc atlas shown in Figure S3 indicates expression in other non-MN cell types in the ventral cord. What is the identity of these neurons.

What role, if any, does Scng play in dI5 neuron differentiation?

First revision

Author response to reviewers' comments

Response to the Reviewer's comments:
Summary:

We were pleased to note that two reviewers were broadly positive about the merit of these studies. Reviewer 1 stated that "*This pseudotime analysis and interpretation provides a valuable resource and characterization of an in vitro differentiation paradigm for generating mouse spinal sensory neurons from mESCs*", while Reviewer 2 felt that "*by compiling pseudotemporal trajectories, [we] were able to gain new insight into the lineage relationships and developmental control of the dorsal horn interneurons and their progenitors.*"

We have responded to the reviews by adding new data, and revising and rewriting the manuscript. In brief, we have added new figures (Figure 4, Figure S3) to compare the dI trajectories derived from our *in vitro* scRNA-Seq data set, with those from a previously generated *in vivo* data set (Delile et al., 2019). We identified broad similarities and some differences, which are both discussed below, and in the manuscript on p13 of the Results section, and p16/17 of the Discussion. We have also added new data to Figure 6 (old Figure 5), which further add support to the hypothesis that we have identified an $Sncg^{+}$ population of dI5s.

Reviewer 1

Advance Summary and Potential Significance to Field:

Similar to previous work from this group, it would be valuable to perform the same pseudotime analysis on mouse embryonic neural tube scRNA-seq data to distinguish and/or confirm their conclusions on developmental branch points and lineage relations from the in vitro system. Without this comparison, it should be made clear (even in the title) that the analysis here is on the in vitro differentiation system.

We thank Reviewer 1 for suggesting this analysis. In the revised version of the manuscript, we now include a comparison of the *in vitro*-derived dI trajectories with an embryonic mouse spinal cord scRNA-Seq dataset from (Delile et al., 2019). The data can be found in Figure 4 and Figure S3. While the *in vivo* dataset is more complex both in time course (E9.5-E13.5 embryos) and in the

© 2024. Published by The Company of Biologists under the terms of the Creative Commons Attribution License (https://creativecommons.org/licenses/by/4.0/).

range of cell types than the *in vitro* data set, there are nonetheless many strikingly similarities with the dI lineage trajectories (Figure 4A, 4B), the convergence of the trajectories as they differentiate, and expression of novel genes (Figure 4C). There are also some differences, which include 1) the lineage of the dI6 population, which segregates with ventral trajectories *in vivo* and 2) the dI3 population, which branches from the dI5s *in vivo*, and the dI2s *in vitro*. 3) It was also not possible to resolve the dI2 lineage relationship. dI2s appear to have a shared lineage with dI4 *in vitro*, whereas *in vivo* it is unclear whether the dI2s branch from a shared dI4/dI5 lineage or the dI1 lineage. This lineage relationship further changes when the *in vitro* and *in vivo* datasets where integrated into a single UMAP (Figure S3) where the dI2 and dI1 trajectories become further apart and do not appear to be derived from a same progenitor pool. We have revised the conclusions of the manuscript to reflect these findings. While the *in vivo* data set is obviously the ground state for this analysis, it is also possible that the complexity of this dataset obscures key lineage relationships at specific time points. These studies should be considered "predictive" and that resolving these lineage relationships will require further lineage tracing/clonal analyses *in vivo*.

Comments for the Author:

<u>Specific comments.</u>

1) As above, are the genes in the branch points and the branch points themselves, the relationship of dI2/3/4, and the new endpoint for dI5 also detected in vivo? A thorough handling of the in vitro scRNA-seq data is included and there are interesting points being made about transitions during dI development. It is easy to forget that these are made on the in vitro system so some rules may not hold in vivo. It should be clear what does hold in vivo and what happens in the culture.

As discussed above, we see that the expression of the different candidate "transition" genes was conserved between *in vitro* and *in vivo* trajectories (Fig. 4C). However, we did see differences in the branch points, which we describe on p13/14 of the manuscript, and discuss on p16/17.

2) Fig 2E is a little misleading (not purposely I'm sure, but from the point of view of a naïve reader) in that it suggests the genes are specific to the different populations but a closer look at Fig S2 suggests that is not that simple (example: Sox4, highlighted in dI5 but is in all the lineages). It is recommended that this point be more explicit when discussing Fig. 2E in describing how these genes are identified and what they mean. Overall however, the way the data are presented for the different dI lineages and the heatmaps provides excellent visuals for what is going on within a given lineage.

We thank Reviewer 1 for appreciating these figures, and have now made clear on p11 that genes identified through the heatmap analysis (Figure 2E) were then evaluated for their trajectory-specific expression (Figure S2) to get more complete picture. Both analyses are necessary - the heatmap analysis is invaluable for identifying enriched expression in trajectories, where patterns can be missed if there is low levels of expression. However, as Reviewer 1 correctly notes, the heatmaps also can give a misleading view of their specificity, and so we have modified the text to note (p11): *"Gene expression was first validated using UMAP plots to determine the extent to which these genes were present in specific trajectories (Fig. S2). We found that the genes were generally expressed in the predicted trajectories, but were not always specific to that lineage."*

3) In the section 'Validation of putative transition state markers in vivo', it would be helpful to put the transitional cluster #s at the beginning sentence where the nine genes came from that were chosen for validation. Along these lines, a supplementary table should be provided showing the top genes (50?, 100?) in each cluster as defined in Fig. 1E since these clusters are used throughout the study. The shinyApp is a useful tool to look through the data but a file with the genes defining the clusters is also needed.

Where appropriate, we have added the cluster identity to this section to better highlight which transitional state being studied for each gene. Specifically, these are: *Gsg1l* (dP1/dI1 - clusters 15/26), *Chrna3* (dP1/dI1 - cluster 15/26), *Cbfa2t2* (dP1/dI1 clusters - 15/26; dP5 – cluster 17), *Smoc1* (dP5- cluster 27), *Gsx1* (dP5 - cluster 27). Four of the selected genes – *Fbxl7, Tfap2b, Prmt8*, and *Sstr2* – are predicted to be present in subsets of multiple dPs as they transition into the

© 2024. Published by The Company of Biologists under the terms of the Creative Commons Attribution License (https://creativecommons.org/licenses/by/4.0/).

intermediate zone based on the pseudotime analysis, and so were present in too many clusters to list. The top 50 genes for each cluster has been added as Table S1.

4) Not all known lineage markers in vivo appear in the in vitro data. One that is discussed a little is Phoxa2. It is suggested the expression is too transient to show up in the data. Another factor notably absent is Ptf1a that is required for the dI4 lineage in vivo. Are Phoxa2 and Ptf1a not in the data because they are too transient? Technical artifacts of scRNAseq? Or is this a difference between the in vitro neural induction versus in vivo development? Clarification through analysis of scRNA-seq from in vivo neural tubes would potentially resolve this.

We see very few *Ptf1a*$^{+}$ and *Phoxa2*$^{+}$ cells in the *in vitro* data set (arrows, Fig. R1A). However – and surprisingly to us – we have subsequently observed that both *Ptf1a* and *Phoxa2* are also expressed in relative few cells in the *in vivo* atlas (arrows, Fig. R1B). The distribution of expression is conserved between the *in vivo* and *in vitro* data sets, thus, *Ptf1a* is present in the dP4 lineage, while *Phoxa2* is present only terminally differentiated dI5 clusters. We attribute these differences to the numbers of sequenced cells, rather than lack of specification: the *in vivo* atlas comprises 38,976 cells, while ~3000 cells were sequenced for the *in vitro* atlas.

Figure R1: Expression of *Ptf1a* and *Phoxa2* in the *in vitro* and *in vivo* atlases.

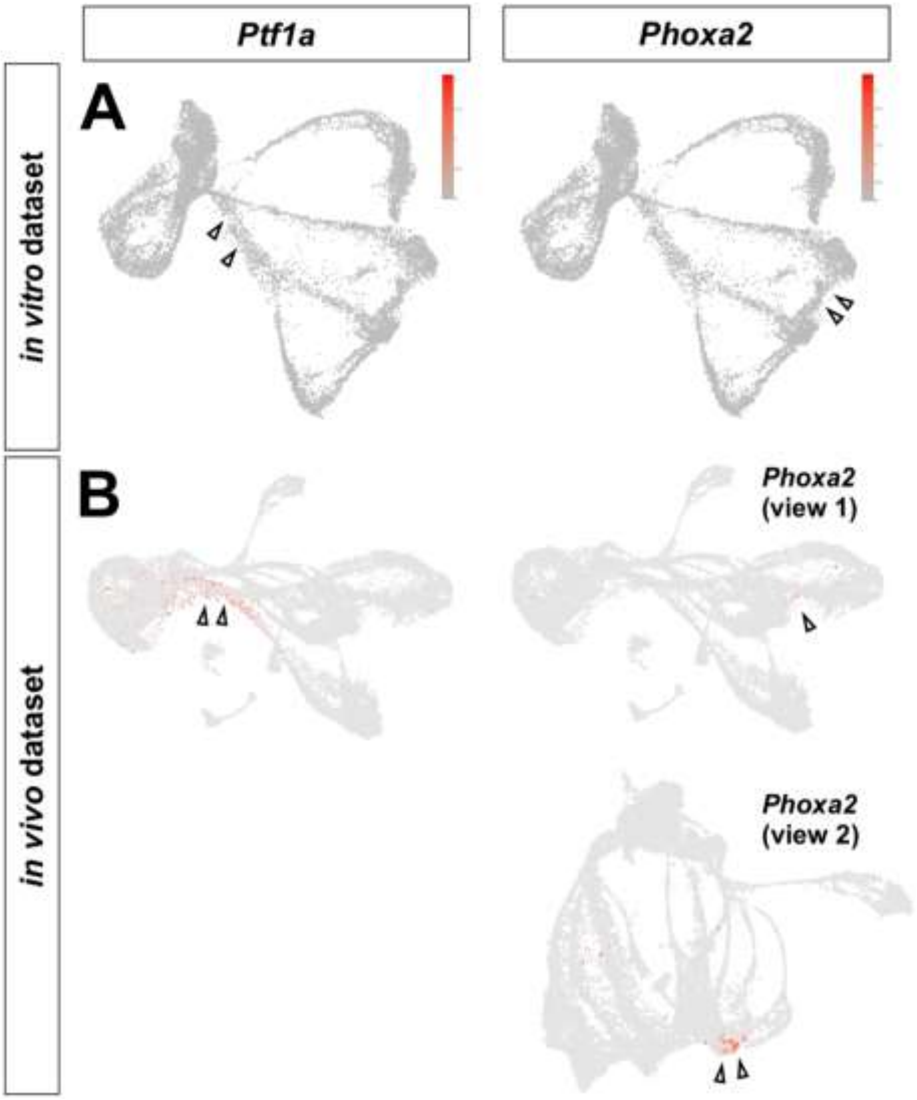

Additional Minor comments:

5) There is no real discussion of what is going on in the progenitor clusters as visualized in Fig. 2C. Are there any insights to come out of this cloud of cells that say anything about progenitor

© 2024. Published by The Company of Biologists under the terms of the Creative Commons Attribution License (https://creativecommons.org/licenses/by/4.0/).

diversity?

We had also hoped that a more detailed analysis of the Sox2^{+} cluster would detect distinct progenitor populations contributing to different dI lineages. However, we were unable to detect these populations, rather we found that the Sox2^{+} population is organized according to different phases of cell cycle, as depicted in Figure R2. We have not included this figure in the paper at this time, but can if Reviewer 1 thinks it sufficiently interesting. We did find that the insulin signaling pathway was specifically expressed in dPs and included this information in the discussion section on page 17 as follows:

"Our analysis of undifferentiated progenitors also reveals novel genes that may regulate dP fate specification. For example, insulin growth factor binding protein (Igfbp) 5, but not Igfbp3, is expressed in cycling spinal progenitors (Fig. 1G), suggesting they respond selectively to insulin signaling. Insulin signaling, acting though Igfbp5, regulates proliferation in other progenitor populations, such the olfactory bulb subventricular zone (Vicario-Abejón et al., 2003) and smooth muscle progenitors (Ahmad et al., 2020; Ren et al., 2008). Thus, modulation of insulin signaling could expand specific dP populations."

Figure R2: Expression of cell cycle genes in progenitor clusters derived in vitro.

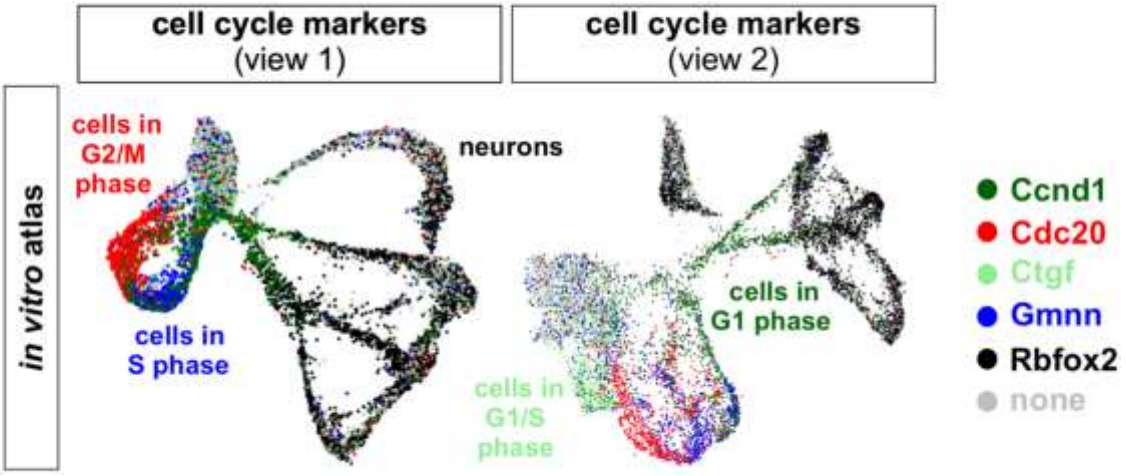

6) Data presentation in figure 3 would be enhanced with co-expression/adjacent known markers for the different populations. Currently, the interpretation of the in situ data must remain imprecise and should be reflected in the way it is described.

We appreciate the suggestion from Reviewer 1, but think it is possible from relatively invariant positions of the dP/dI domains which have been thoroughly described in the literature, to get a general sense of the identities, especially the dP1/dI1 domains. That said, we have revised the text to be more careful not to overinterpret these expression patterns.

7) Suggestion: Figure 5 shows nice co-staining of Sncg with Lmx1b and Pax2. The high mag images shown for the triple are clear. If possible, it is suggested to do the same for Sncg/Lmx1b and Sncg/Pax2 rather than the lower mag images.

We have revised Figure 6 (old Figure 5), adding new panels showing co-labeling of Lmx1b and Sncg antibodies, at high and low magnification (Figure 6C, 6D). This new analysis demonstrates that the dorsal-most population of Sncg^{+} population continues to also be Lmx1b^{+}, and appears to be migrating to a more superficial layer, consistent with what has been previously shown for dI5s (Roome et al., 2020).

8) On pg 16 in the discussion, it is not clear how the few sentences on Tfap2b and Ptf1a fits with the point of the paragraph. Maybe integrate or delete?

We apologize for this confusion. This section was meant to highlight the finding that *Tfap2b* has

© 2024. Published by The Company of Biologists under the terms of the Creative Commons Attribution License (https://creativecommons.org/licenses/by/4.0/).

been shown to work downstream of *Ptf1a* in other contexts, such as cerebellum development, and the same regulatory context could exist in the spinal cord as well.

We have modified this section on p18 to read as follows: *"Similarly, there is evidence of regulatory interactions between Tfap2b and Ptf1a in other neuronal contexts. For example, Tfap2b expression is regulated by Ptf1a during cerebellar and retinal neurogenesis (Jin et al., 2015; Zainolabidin et al., 2017). In the spinal cord, Ptf1a is expressed specifically in the dP4 domain, suggesting it may activate Tfap2b to regulate dI4/dI6 differentiation."*

We hope that this revision makes this discussion point clearer.

Reviewer 2

Advance Summary and Potential Significance to Field:

Comments for the Author:

1) Is this surprising for inhibitory dI4 neurons and excitatory dI2 and dI3 to share the same lineage relationship during early development?

We found it surprising too. However, unlike the canonical view of cortical development where excitatory and inhibitory neurons arise from different progenitors, these populations can be derived from the same progenitor domain in the spinal cord. For example, inhibitory dI4s and excitatory dI5s both arise from the $Ascl1^{+}$ population. Interactions between the Ascl1, Prdm13 and Ptf1a transcription factors then determine lineage specification (Chang et al., 2013; Mona et al., 2017). The new *in silico* analysis of *in vivo* scRNA-Seq dataset also supported their common lineage - since we observed the dI4s and dI5s branching off from a common pool of progenitors, along with the dI3s (excitatory). This paradigm has also been seen in other contexts, including mouse cerebellar development (Zhang et al., 2021) and recent observations from the human cortex have suggested that human cortical progenitors can produce both excitatory and inhibitory neurons (Delgado et al., 2022).

2) Related to the above points, the authors try to claim that at later stages, dI2-6 neurons start to share similar transcriptomes again. This again appears not to make much sense since you are talking about excitatory (DI2, DI3, dI5) versus inhibitory neurons (dI4, dI6). It is possible that this in vitro differentiation protocol may not capture the later terminal differentiation seen in vivo. Can the authors comment on the available transcriptomes of adult dorsal horn interneurons that correspond to these embryonic dorsal horn interneurons? The authors need to have some serious discussions on these points.

While the transcriptional convergence of diverse neural cell types might seem counterintuitive, we have now observed the same phenomenon in *in vivo* scRNA-seq data set derived from (Delile et al., 2019) (Figure 4 B). With the notable exception of the dI3s and MNs, the dorsal and ventral interneurons *in vivo* show convergence to a more transcriptionally similar state, suggesting that the *in vitro* protocol accurately captured a feature of dI terminal differentiation. These *in vivo* results are now described on p13.

Other examples can be found in the literature. In a recent scRNA-Seq profiling of developing mouse DRGs, there is similar transcriptional convergence at the end of sensory neuron differentiation, which correlated with the emergence of diverse subtypes (Faure et al., 2020). Another study examining scRNA-Seq lineages in developing C. elegance embryos, reported similar transcriptional convergence at the end of multiple co-fated lineages (Packer et al., 2019). Together, these studies suggest that transcriptional heterogeneity decreases in differentiating neural subtypes that share largely similar functional properties. As requested by Reviewer 2, we have added further discussion on this point on p17/18 in the revised version of the manuscript.

Reviewer 3

© 2024. Published by The Company of Biologists under the terms of the Creative Commons Attribution License (https://creativecommons.org/licenses/by/4.0/).

Advance Summary and Potential Significance to Field:

This study adds little to what is already known about neuronal specification in the dorsal spinal cord, and it has multiple weaknesses that need addressing. In particular, the proposed trajectories require further analysis and validation. A major rewrite will also be required before it is suitable for publication.

We respectfully disagree with Reviewer 3's assessment. This study opens up many new avenues, from validating a stem cell-based model system for understanding spinal cord development and establishing a platform to identify novel genes that are putative regulators of sensory circuit development. Moreover, the data has been validated using multiple approaches, including (1) pseudotime analyses, (2) a direct comparison with an *in vivo* scRNA-Seq data set (new Fig. 4) which demonstrates strikingly similarities in the lineage trajectories and (3) *in situ* hybridization analyses of novel "transition" genes identified through the *in vitro* trajectory analysis. In 8/9 cases the predicted expression pattern of these genes was observed *in vivo*. These studies, together with the new tools we have generated, are thus likely to be considerable interest to the field.

Comments for the Author:

1) The authors have used an in vitro ES cells differentiation protocol to analyze the developmental trajectories of dorsal spinal cord progenitors. They draw a number of conclusion from their bioinformatic analysis, namely that the dI5 interneuron progenitor population, followed by dI1 progenitors are the first to branch off a common dorsal progenitor lineage, with dI2/3/4 progenitors forming a common late lineage that then separates into three lineages. The authors identify a number of genes that mark this transition, although the rationale for choosing these genes and the function of these genes is in many cases is not adequately discussed. There is some concern with the the proposed trajectories for dI1-dI6 neurons, and how these trajectories comport with previously published in vivo analyses at these early embryonic stages. Further in vivo validation of these trajectories is needed, potentially by comparing their in vitro analysis with a similarly performed dorsal spinal cord single cell RNASeq analysis at times spanning the early differentiation of dorsal spinal cord cell types, i.e. E9-E11.

We thank Reviewer 3 for this suggestion. In the revised version of the manuscript, we have now compared the in vitro-derived dI1 trajectories with an *in vivo* scRNA-Seq dataset taken from E9.9-E13.5 spinal cords (Delile et al., 2019). While this comparison revealed the considerable similarities between the *in vitro* and *in vivo* data (new Fig, 4), both at the level of cell types and developmental progression, there were also differences in the lineage relationships of the dI trajectories, as discussed above in first comment to Reviewer 1. This data is presented on p13 of the results section, and discussed on p16/17. We have revised the conclusions of the paper to reflect these changes, and note that further *in vivo* lineage tracing studies are needed to resolve these questions.

We have added further rationale for choosing genes identified in our trajectory analysis. As stated on p11, we sought to identify genes that establish transition states that drive the specification of the dI trajectories, using the heat map and UMAP analysis. We focused on the genes expressed in key transitions that had not been previously analyzed (*Fbxl7, Prmt8, Cbfa2t2, Tfap2b, Chrna3, Smoc1, Sstr2*) as well as some known genes (*Gsg1l, Gsx1*). As described in the text, and in Figure 3, *Gsg1l*, *Cbfa2t2*, and *Chrna3* were predicted to be expressed in the dP1/dI1 lineage, while Smoc1 and Gsx1 were predicted to be expressed in the dP5 lineage. The other four genes were all predicted to be present in the intermediate zone. Our analysis strikingly confirmed these predictions for 8/9 genes, suggesting this data set will be a useful tool for further discovery.

2) I would also argue for a far more scholarly reading and citing of the literature as many critical studies were not cited. Moreover, in some instances these studies suggest alternate trajectories and developmental decision points. For example, the functional analysis of Ptf1a (Glasgow et al., 2005) argues for a common progenitor for dI4 and dI5 neurons, and there is evidence that at E10.5 Lmx1b dI5 neurons are born after Pax2+ dI5 neurons have already been generated. There is evidence for Notch signaling controlling the fate of dI4 versus dI5 neurons. While the findings in this study may be correct, this needs to validated and then discussed in the context of the published literature. For example, there needs to be a fuller discussion of

© 2024. Published by The Company of Biologists under the terms of the Creative Commons Attribution License (https://creativecommons.org/licenses/by/4.0/).

genes such as Atoh1, Ngn1/2, Olig3, Ascl1, Lbx1, Ptf1, Notch and Gbx1/2 that have demonstrated roles in dorsal cell specification.

We appreciate the Reviewer 3 desire to be thorough, and have now added further references to the introduction on p3, including a brief summary of role of the key transcription factors. However, because of space limitations, we have to also refer the readers to some of the reviews on dorsal cell specification written by ourselves (Andrews et al., 2019; Gupta and Butler, 2021) and others (Lai et al., 2016) for a fuller discussion of the critical regulatory genes. We would be happy to add any further studies/reviews as Reviewer 3 requires.

3) I also have some concerns with the analysis of Sncg. The authors show expression at E11.5, which is quite late, as dI5 neurons are first generated at E10-E10.5. Better panels showing co-expression with Lmx1b is needed (Figure 5) as it is hard to ascertain whether these two genes are co-expressed in dI5 cells. The expression of Scng from the Allen sc atlas shown in Figure S3 indicates expression in other non-MN cell types in the ventral cord. What is the identity of these neurons. What role, if any, does Scng play in dI5 neuron differentiation?

We thank Reviewer 3 for pointing it out these concerns. We have revised Figure 6 (old Figure 5) by adding immunohistochemical data for Lmx1b and Sncg at stages E12.5/E13.5. We have found that the dorsal-most Sncg^{+} population co-expresses Lmx1b^{+} at both E12.5 and E13.5, and moreover, appears to be migrating to a more superficial layer, as previously shown for dI5s (Roome et al., 2020). We appreciate the request to understand the role of Sncg in the developing and adult spinal cord, however this analysis is outside the scope of this current study.

References:

Ahmad, S.S., Ahmad, K., Lee, E.J., Lee, Y.H., Choi, I., 2020. Implications of Insulin-Like Growth Factor-1 in Skeletal Muscle and Various Diseases. Cells 9.

Andrews, M.G., Kong, J., Novitch, B.G., Butler, S.J., 2019. New perspectives on the mechanisms establishing the dorsal-ventral axis of the spinal cord. Current topics in developmental biology 132, 417-450.

Chang, J.C., Meredith, D.M., Mayer, P.R., Borromeo, M.D., Lai, H.C., Ou, Y.H., Johnson, J.E., 2013. Prdm13 mediates the balance of inhibitory and excitatory neurons in somatosensory circuits. Dev Cell 25, 182-195.

Delgado, R.N., Allen, D.E., Keefe, M.G., Mancia Leon, W.R., Ziffra, R.S., Crouch, E.E., Alvarez-Buylla, A., Nowakowski, T.J., 2022. Individual human cortical progenitors can produce excitatory and inhibitory neurons. Nature 601, 397-403.

Delile, J., Rayon, T., Melchionda, M., Edwards, A., Briscoe, J., Sagner, A., 2019. Single cell transcriptomics reveals spatial and temporal dynamics of gene expression in the developing mouse spinal cord. Development 146.

Faure, L., Wang, Y., Kastriti, M.E., Fontanet, P., Cheung, K.K.Y., Petitpre, C., Wu, H., Sun, L.L., Runge, K., Croci, L., Landy, M.A., Lai, H.C., Consalez, G.G., de Chevigny, A., Lallemend, F., Adameyko, I., Hadjab, S., 2020. Single cell RNA sequencing identifies early diversity of sensory neurons forming via bi-potential intermediates. Nature communications 11, 4175.

Gupta, S., Butler, S.J., 2021. Getting in touch with your senses: Mechanisms specifying sensory interneurons in the dorsal spinal cord. WIREs Mech Dis 13, e1520.

Lai, H.C., Seal, R.P., Johnson, J.E., 2016. Making sense out of spinal cord somatosensory development. Development 143, 3434-3448.

Mona, B., Uruena, A., Kollipara, R.K., Ma, Z., Borromeo, M.D., Chang, J.C., Johnson, J.E., 2017. Repression by PRDM13 is critical for generating precision in neuronal identity. Elife 6.

Packer, J.S., Zhu, Q., Huynh, C., Sivaramakrishnan, P., Preston, E., Dueck, H., Stefanik, D., Tan,

© 2024. Published by The Company of Biologists under the terms of the Creative Commons Attribution License (https://creativecommons.org/licenses/by/4.0/).

K., Trapnell, C., Kim, J., Waterston, R.H., Murray, J.I., 2019. A lineage-resolved molecular atlas of. Science 365.

Ren, H., Yin, P., Duan, C., 2008. IGFBP-5 regulates muscle cell differentiation by binding to IGF-II and switching on the IGF-II auto-regulation loop. J Cell Biol 182, 979-991.

Roome, R.B., Bourojeni, F.B., Mona, B., Rastegar-Pouyani, S., Blain, R., Dumouchel, A., Salesse, C., Thompson, W.S., Brookbank, M., Gitton, Y., Tessarollo, L., Goulding, M., Johnson, J.E., Kmita, M., Chedotal, A., Kania, A., 2020. Phox2a Defines a Developmental Origin of the Anterolateral System in Mice and Humans. Cell reports 33, 108425.

Vicario-Abejón, C., Yusta-Boyo, M.J., Fernández-Moreno, C., de Pablo, F., 2003. Locally born olfactory bulb stem cells proliferate in response to insulin-related factors and require endogenous insulin-like growth factor-I for differentiation into neurons and glia. J Neurosci 23, 895-906.

Zhang, T., Liu, T., Mora, N., Guegan, J., Bertrand, M., Contreras, X., Hansen, A.H., Streicher, C., Anderle, M., Danda, N., Tiberi, L., Hippenmeyer, S., Hassan, B.A., 2021. Generation of excitatory and inhibitory neurons from common progenitors via Notch signaling in the cerebellum. Cell Rep 35, 109208.

Second decision letter

MS ID#: DEVELOP/2023/202209

MS TITLE: Investigating the basis of lineage decisions and developmental trajectories in the dorsal spinal cord through pseudotime analyses

AUTHORS: Sandeep Gupta, Eric Heinrichs, Bennett G Novitch, and Samantha J Butler
ARTICLE TYPE: Research Article

I am delighted to tell you that your manuscript has been accepted for publication in Development, pending our standard ethics checks. Please not that referee 1 has noticed a typo in your revised manuscript (a reference for Fig. 4F while there is no Figure 4 panel F).

Reviewer 1

Advance summary and potential significance to field

My comments/suggestions are all addressed in this revision. Adding the analysis and comparison to the in vivo neural tube scRNA-seq data substantially improved the study.

Comments for the author

One typo--cites Fig. 4F at one point but there is no 4F.

Reviewer 2

Advance summary and potential significance to field

The study provides new information on lineage relationships among different spinal neurons.

Comments for the author

© 2024. Published by The Company of Biologists under the terms of the Creative Commons Attribution License (https://creativecommons.org/licenses/by/4.0/).

The authors have carefully addressed my comments. The newly added comparisons with in vivo scRNAseq data are informative, showing the in vitro differentiation paradigm can recapture in vivo lineage relationships but also indicate some clear differences.

© 2024. Published by The Company of Biologists under the terms of the Creative Commons Attribution License (https://creativecommons.org/licenses/by/4.0/).
